# Supplementary figures and images for: The effect of UVA light/8-methoxypsoralen exposure used in Extracorporeal Photopheresis treatment on platelets and extracellular vesicles
Source: PLoS One. 2024 Feb 28;19(2):e0293687. doi: 10.1371/journal.pone.0293687 (PMC10901342; doi:10.1371/journal.pone.0293687)

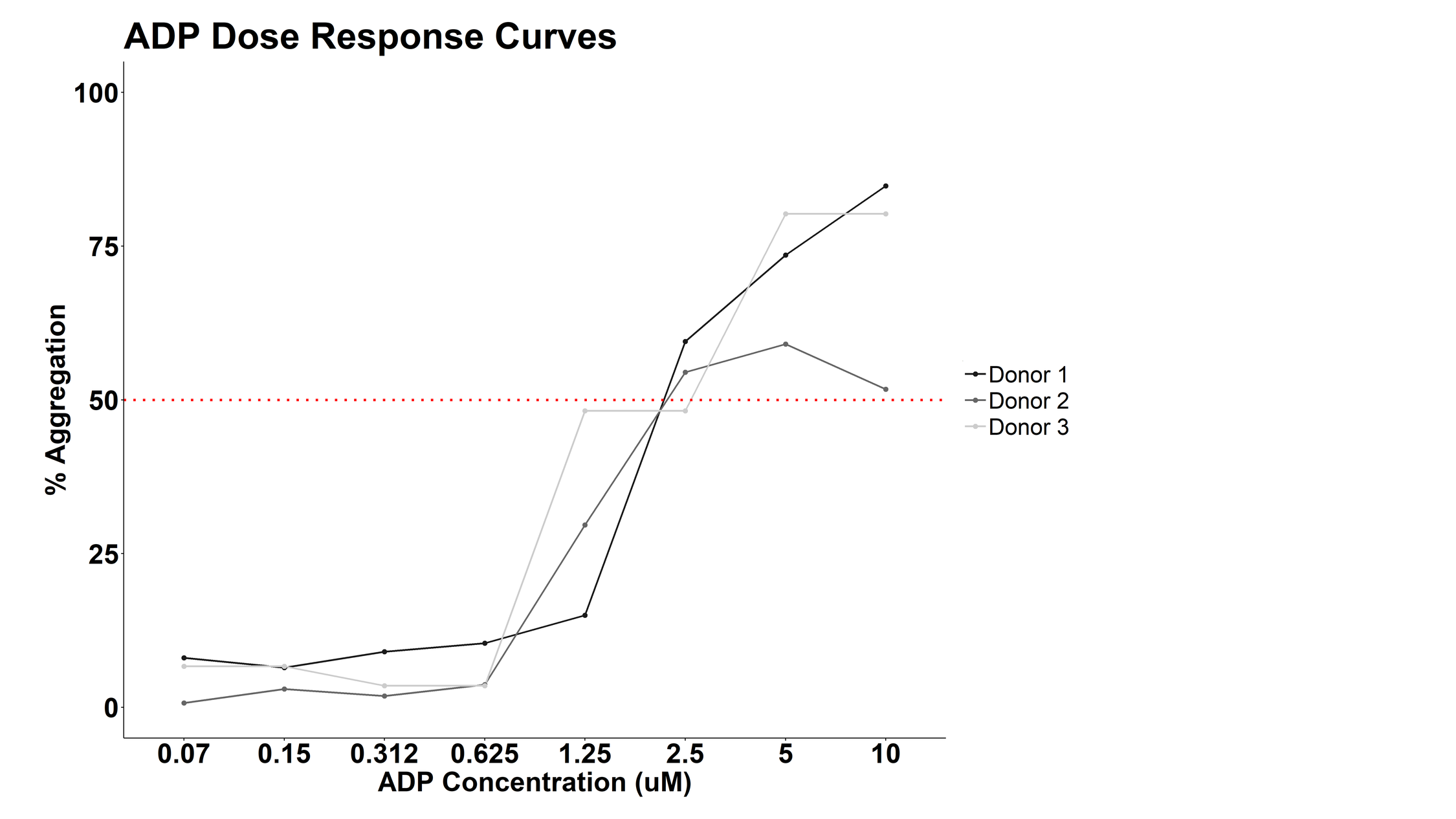

Supplement: S1 Fig — PRP was isolated from 3 healthy volunteers to carry out an adenosine diphosphate (ADP) dose response curve using the 96-well plate aggregometry assay. ADP concentrations from 0.07 μM to 10 μM ADP were assessed. 1.25 μM ADP resulted in platelet aggregation <50% aggregation (marked with a red dotted line) and was used for all follow-on experiments. (TIF) [file pone.0293687.s001.tif]

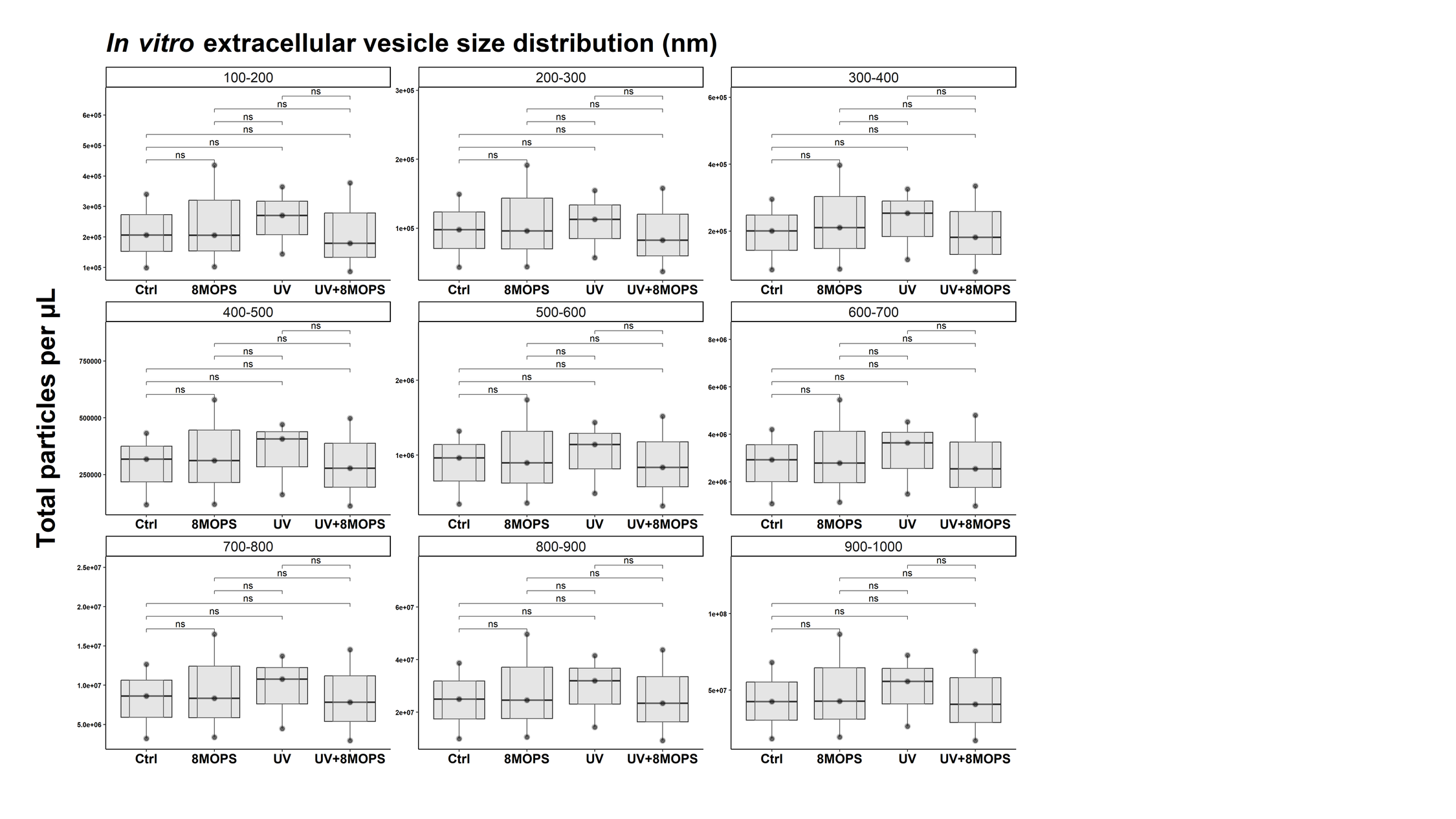

Supplement: S2 Fig — Flow Cytometry was performed with the CytoFlex S to quantify large EVs (100-1000nm) in PPP post-exposure to UVA light and/or 8-methoxypsoralen. Light scatter intensities were adjusted to reflect biological EV properties using Rosetta calibration beads and software. Large EV size remained unchanged upon dual and individual UVA light/8-methoxypsoralen exposure. 8MOPS: 8-methoxypsoralen. (TIF) [file pone.0293687.s002.tif]

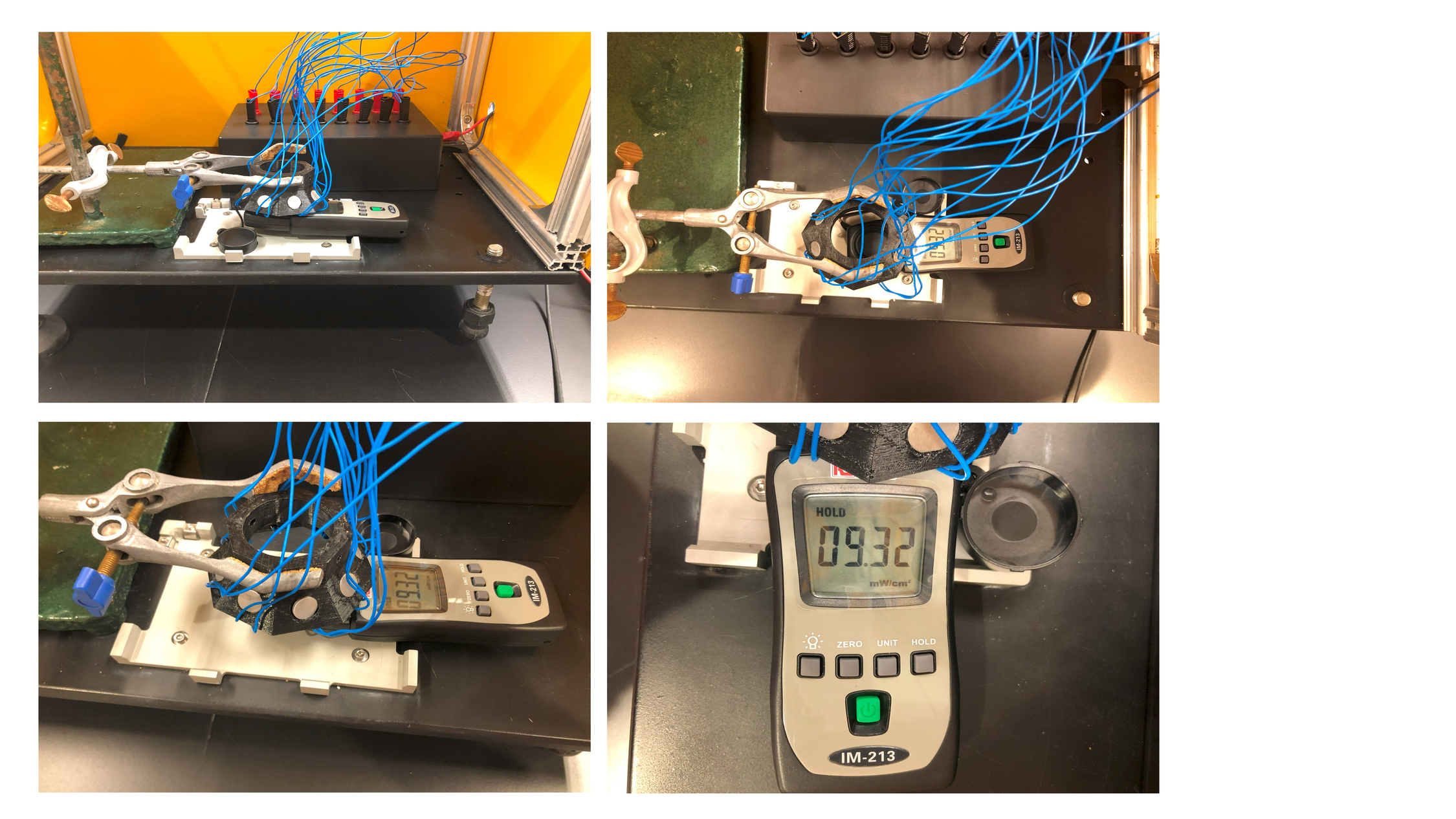

Supplement: S3 Fig — A UVAB light meter (mW/cm2) was used to ensure the correct wavelength and intensity of light used to echo the ECP dose. The RS Pro UVAB light meter measures UV light in the range of 290-390nm, encapsulating the ECP UVA light range of 320-400nm. An average of 10mW/cm2 UV intensity was recorded at the lowest stage hight of this UV light box and using the equation Watt x Time = Joules gives you 2 min and 30 sec of this UV intensity to supply 1.5 Joules/cm2 UVA light to the samples in a 96-well plate. (TIF) [file pone.0293687.s003.tif]
